# Longitudinal changes of choroid plexus volumes and MRI ratios in multiple sclerosis

**DOI:** 10.1093/braincomms/fcag129

**Published:** 2026-04-18

**Authors:** Britta Krieger, Barbara Bellenberg, Dajana Müller, Hasan Sbaihat, Anna Katharina Roenneke, Theodoros Ladopoulos, Ruth Schneider, Ralf Gold, Carsten Lukas

**Affiliations:** Ruhr University Bochum, St. Josef Hospital, Institute of Neuroradiology, 44791 Bochum, Germany; Ruhr University Bochum, St. Josef Hospital, Institute of Neuroradiology, 44791 Bochum, Germany; Ruhr University Bochum, St. Josef Hospital, Institute of Neuroradiology, 44791 Bochum, Germany; Ruhr University Bochum, St. Josef Hospital, Institute of Neuroradiology, 44791 Bochum, Germany; Ruhr University Bochum, St. Josef Hospital, Institute of Neuroradiology, 44791 Bochum, Germany; Ruhr University Bochum, St. Josef Hospital, Department of Neurology, 44791 Bochum, Germany; Ruhr University Bochum, St. Josef Hospital, Department of Neurology, 44791 Bochum, Germany; Ruhr University Bochum, St. Josef Hospital, Department of Neurology, 44791 Bochum, Germany; Ruhr University Bochum, St. Josef Hospital, Institute of Neuroradiology, 44791 Bochum, Germany; Ruhr University Bochum, St. Josef Hospital, Department of Neurology, 44791 Bochum, Germany

**Keywords:** multiple sclerosis, brain MRI, choroid plexus, longitudinal, lesions

## Abstract

This longitudinal study aimed to identify the predictive role of choroid plexus enlargement for disability progression and its association with tissue damage in early multiple sclerosis patients. Forty patients with clinically isolated syndrome or early relapsing–remitting multiple sclerosis underwent brain MRI scanning at baseline, after 2 and after 6–8 years. FreeSurfer segmentation was used for brain volume estimation, and the choroid plexus was segmented by use of an independent automated segmentation tool. MRI ratios (T1 weighted through fluid attenuated inversion recovery image values) were calculated after calibration and subtracting lesion masks. Lesion masks were eroded to obtain both lesion rims and centres, for which mean ratios were extracted in addition to normal-appearing white matter. Expanded Disability Status Scale and disease duration were collected from neurological examination. Between baseline and last follow-up, choroid plexus volumes increased, and MRI ratios in lesions decreased significantly, which was dominated by the decrease in clinically isolated syndrome rather than relapsing–remitting patients and more pronounced in lesion rims than in their centre. Choroid plexus volumes were significantly related to MRI ratios within rims of lesions at baseline and within lesion centres after 6–8 years, but not within normal-appearing white matter. No associations between choroid plexus volumes or ratios and disability were found. We observed longitudinal changes of both choroid plexus enlargement and MRI ratios over 6–8 years in early multiple sclerosis patients. Choroid plexus enlargement seemed involved in the dynamic development of lesion destructivity.

## Teaser

Krieger *et al*. report longitudinal changes of two promising brain MRI biomarkers in early multiple sclerosis patients: choroid plexus volumes increased and lesional tissue integrity assessed by ratios of T1-weighted to fluid-attenuated inversion recovery MRI decreased and were associated with each other.

## Graphical Abstract

**Graphical Abstract:**
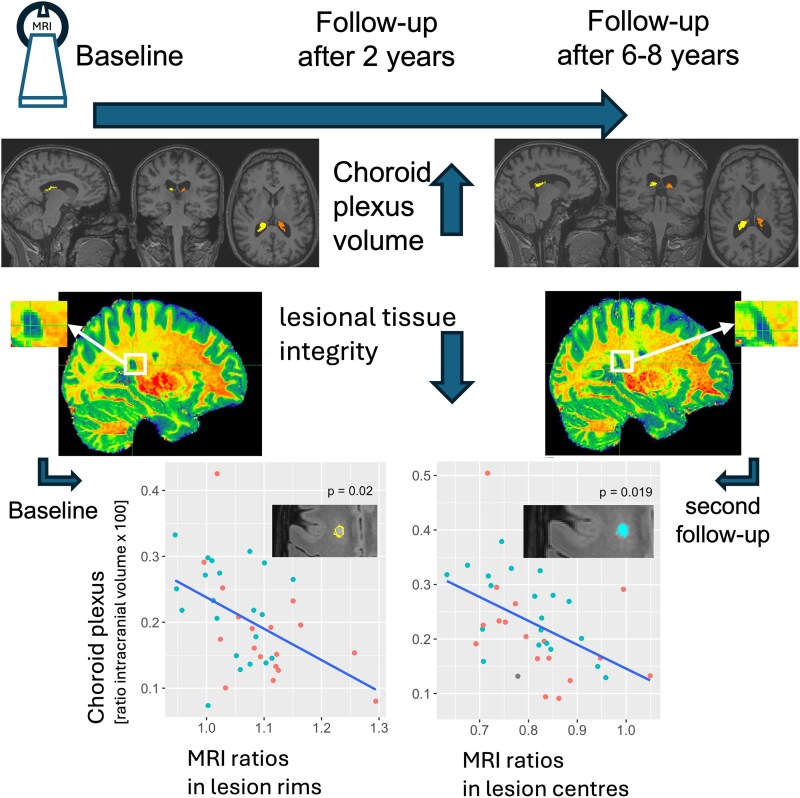

## Introduction

In recent years, the choroid plexus (ChP) has been suggested to be a marker for neuroinflammation as it is responsible for regulation of immune cells and other molecules that enter the CNS at the blood–CSF barrier (BCSFB). In multiple sclerosis (MS), pro-inflammatory immune cells entering the CNS from peripheral regions can trigger disease-specific inflammatory cascades.^1^ Thus, acute inflammatory processes in neurological diseases, such as MS, might be studied by investigating ChP alterations.^2,3^ Furthermore, the ChP has been shown to be enlarged and to be associated with disease activity in MS.^4^ Not only neuroinflammation but also microstructural integrity loss and demyelination represent a hallmark of MS pathology. However, it is unclear how these processes are related to each other and how the ChP enlargement is involved in this pathology.

Tissue damage, including myelin loss, axonal degradation or dendrite density reduction in the brain, has been shown to be identifiable by MRI biomarkers, such as T1-weighted (T1w) to T2-weighted (T2w) ratios.^5-7^ However, fluid-attenuated inversion recovery (FLAIR) image acquisition has been evolved as a standard in clinical routine rather than T2w since lesions are nowadays detected on FLAIR images.^8^ Therefore, T1w/FLAIR ratios have been suggested as a more suitable and standardizable biomarker that might serve as a biomarker for tissue integrity similar to T1w/T2w ratios.^9^

The development of demyelinating and neuroinflammatory processes over time and their relationship remain to be elucidated. In our previous cross-sectional study, we were able to combine both ChP volumes and T1w/FLAIR ratios as we showed that ChP enlargement was present in relapsing–remitting MS (RRMS) patients compared to healthy controls and that microstructural tissue damage in normal-appearing white matter (NAWM) could be assessed by reduced T1w/FLAIR ratios in patients than in controls.^10^ Both processes might be relevant for tracking disease progression over time and might especially provide evidence for lesion development. In the present study, we assessed the longitudinal alterations of ChP volume and T1w/FLAIR ratios in the brain in a cohort of patients with early MS or clinically isolated syndrome (CIS) from the time of diagnosis to 8 years of follow-up. This very early stage of the disease is of particular interest, as early treatment decisions can have an impact on the subsequent course of the disease.

The main aims of this study were 2-fold. On the one hand, we aimed to identify the predictive role of ChP enlargement for disability progression in early MS and CIS. On the other hand, we aimed to study the interaction between neuroinflammation and microstructural integrity in the early phases of the disease by longitudinal assessment of both ChP enlargement and T1w/FLAIR ratio development in detail.

## Materials and methods

### Patients

In our monocentric study, we included patients who have taken part in the prospective longitudinal German National MS Cohort Study (NationMS) of the German Competence Network Multiple Sclerosis (KKNMS), for which patients have been recruited since 2010. According to the study design, the enrolled patients were disease-modifying treatment (DMT) naïve prior to inclusion. All patients fulfilled the diagnosis of CIS within the 6 months prior to inclusion, or early definite RRMS based on the McDonald 2010 criteria.^11^ As the baseline assessment took place before 2017, the classification followed the diagnostic McDonald criteria of 2010 and did not take into account information on oligoclonal bands (OCBs) as in the revised McDonald criteria.^12^ However, additional reclassification by collection of lumbar puncture results was conducted to discuss classification differences.

### Magnetic resonance imaging acquisition parameters

Imaging was performed using a standardized MRI brain imaging protocol on a 3 T Philips Achieva scanner with a 32-channel phased-array head coil. The MRI protocol included high-resolution, isotropic three-dimensional (3D) T1-weighted (3D-T1w, repetition time, 0.01 s; echo time, 4.6 ms; voxel size, 1 × 1 × 1 mm^3^; field of view, 240 mm) and 3D-fluid-attenuated inversion recovery/(FLAIR, repetition time, 4.8 s; echo time, 293 ms; inversion time, 1.65 ms; flip angle, 90°; turbo factor, 182; voxel size, 1 × 1 × 1 mm^3^; field of view, 240 mm) sequences with a field of view covering the brain and the upper cervical cord. The vendor-specific 3D distortion correction procedures were used to correct for non-linear gradient distortion effects.^13^ Contrast-enhancing lesions were identified after gadolinium administration on T1w images.

### Image processing

T1w and FLAIR images were processed using Samseg to obtain white matter lesion masks and FreeSurfer’s reconstruction pipeline to receive total brain volumes, lateral ventricle volumes (LVVs) and white matter segmentation masks.^14,15^ Consistency of image quality between the three time points was assessed by the contrast signal-to-noise (conSNR) values from FreeSurfer’s quality assurance tool (results are summarized in the Supplementary material under Supplementary Section 2).

ChP segmentations were derived from an automated segmentation procedure.^16^ An example is depicted in the Supplementary material (Supplementary Fig. 3). Total lesion volumes (TLVs) were obtained by using the lesion prediction algorithm (LPA) implemented in the lesion segmentation toolbox (LST), part of the Statistical Parametric Mapping software (SPM).^17^

The registered T1w and FLAIR images were further processed based on a method described by Cappelle *et al*.^9^ to acquire T1w/FLAIR ratios. These authors demonstrated the feasibility of T1w/FLAIR ratios as a biomarker for tissue integrity in MS and compared different methods for image calibration to account for between-subject and within-subject intensity variations. The authors concluded that non-linear image calibration methods produced the best results; therefore, we chose their non-linear histogram calibration. In this method, a non-linear histogram matching is performed by first calculating an intensity histogram from the extra-cerebral tissue of a template and then applying the histogram transformation to the T1w or FLAIR images to obtain calibrated images. In this step, we excluded the lesion masks from the brain tissue masks that were used for calibration, to reduce the impact of lesion voxels on the calibrated ratios. *Mrcalc* was used to calculate the T1w/FLAIR ratios.^18^

Median T1w/FLAIR ratios were calculated within NAWM and lesions using *mrstats.*^18^ Lesion masks were eroded by 1 mm to receive lesion rim masks. Robustness of results was assessed by applying different erosion thresholds of 1.5 and 2 mm (see Supplementary Section 3 and Supplementary Figure 1).

We checked the quality of the T1w/FLAIR images by visual inspection for any artefacts or imperfect registration for all MRI sessions. The correctness of lesion mask and ChP segmentations was confirmed by visual inspection for all participants at each of the three time points. We evaluated the lesion and ChP segmentations by overlaying them on the corresponding 3D-FLAIR and 3D-T1w series, respectively. As the lesion rim masks were derived from lesion segmentation, they were only checked exemplarily. No corrections were made as the quality assessments were satisfactory in terms of visible false negatives or false positives, or T1w/FLAIR artefacts. In addition, no inter-rater metrics were available.

### Clinical parameters

Disease duration, defined at study inclusion (baseline) as the time of symptom onset, relapses and Expanded Disability Status Scale (EDSS) data were acquired from the electronic health record system. The disease course was classified by the treating physician at baseline (ses0) and at follow-up visits after 2 years (ses1) and 6–8 years (ses2) as RRMS or CIS. In addition, the development of secondary progressive MS (SPMS) was classified based on the clinical presentation assessed by the treating physician, including observation of clinical worsening, regardless of individual relapses, over at least 6 months. EDSS was determined by experienced neurologists to assess clinical disability in MS based on several functional systems. EDSS progression between ses0 and ses2 was classified as worsened for an increase by ≥1.5 when baseline EDSS was 0, for an increase by ≥1.0 when EDSS at baseline was <6.0 and for an increase by ≥0.5 when EDSS at baseline was ≥6.0.^19^ At study entry, all patients were treatment naïve, but a DMT might have been initiated after baseline acquisition, which was divided into low- (interferon, fumarate, glatiramer acetate, azathioprine) and high-efficacy treatment (natalizumab, fingolimod, rituximab, ocrelizumab, Fumaderm, Mavenclad).^20^

### Statistical analysis

Statistical analysis was performed with R software (version 4.0.1).^21^ Bilateral ChP volumes were calculated by summing both hemispheres, which were then normalized to intracranial volume and multiplied by 100 before further analyses.

Linear mixed models were calculated to analyse longitudinal changes of lesion volumes, EDSS, T1w/FLAIR ratios or ChP volumes and their differences between CIS and RRMS groups using age at baseline and sex as fixed effects and subjects as random effects. The function *lmer* was used, which omits any missing value from model estimation; i.e. it strips any observations with any missing values in any variables.^22^ Pairwise comparisons were conducted using the *emmeans* package in R,^21^ which uses the Tukey method for multiple comparison *P*-value adjustment. In order to reduce a potential bias due to a strong relationship between ChP and LVVs, which has been discussed in the literature recently, we have conducted a second analysis adding also LVV as a covariate of no interest in the linear mixed models.

Linear regression analyses with age and ventricular volume as covariates were conducted to analyse the relationship between ChP volumes at baseline and EDSS, EDSS progression or lesion volumes. Linear regression analyses were further used to examine the association between ChP volumes and T1w/FLAIR ratios in NAWM or lesion rims/centres with age at baseline and sex as covariates. Annualized ChP volume change rates were calculated for the first follow-up period (from ses0 to ses1) and the second follow-up period (from ses1 to ses2).

### Standard protocol approvals, registrations and patient consents

The study was approved by the local ethics committee of Ruhr University Bochum (approval no. 3714-10), and all patients provided written informed consent prior to study participation.

## Results

### Demographics

**Figure 1:**
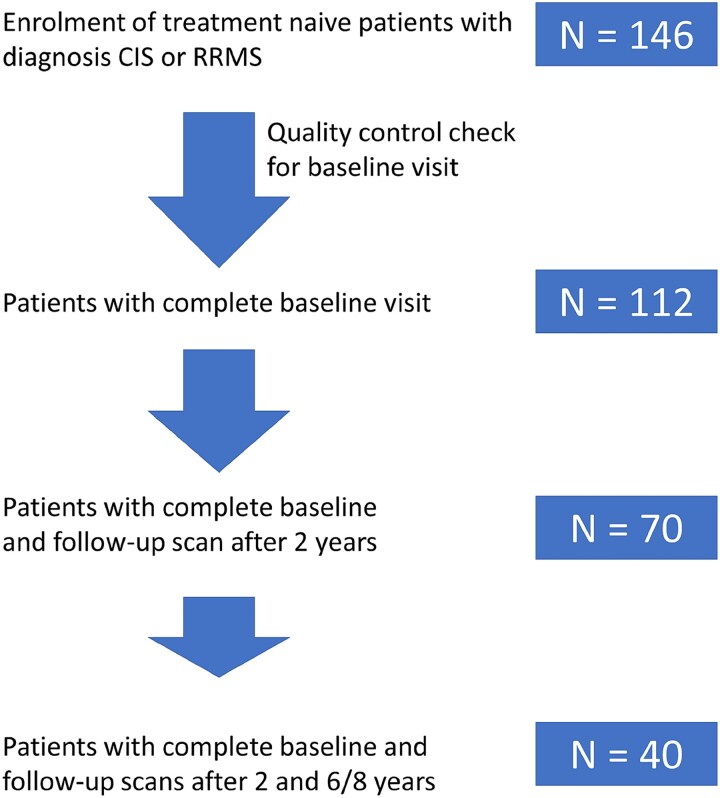
**Data exclusion procedure.** Flow diagram for data exclusion procedure.

From the total number of patients enrolled in our local KKNMS cohort (*N* = 146), the presented study included 40 patients who completed MRI scanning at baseline, after 2 years and after 6–8 years (Fig. 1). Reasons for exclusions were unavailability, interruption of participation, lack of data or withdrawal from the study. Demographical data was summarized in Table 1. Overall, patient’s disability measured by EDSS was stable over time (*P*-value for each time period >0.9). Three patients converted to SPMS at the last follow-up.

**Table 1:** Demographical data

Characteristic *N* = 40	ses0 (baseline)	ses1 (2 years)	ses2 (6–8 years)
Sex			
Female	21 (53%)	21 (53%)	21 (53%)
Male	19 (48%)	19 (48%)	19 (48%)
Age	38 (13)		
Age at disease onset	37 (13)		
Disease duration (years since symptom onset)	0.64 (0.64)		
Follow-up time (months)		24 (3)	73 (9)
EDSS	1.96 (1.12) [0.0, 5.0]	1.96 (1.30) [0.0, 6.5]	2.04 (1.53) [0.0, 6.0]
*Unknown*			*2*
Patients with EDSS progression		8	11
*Unknown*			*2*
Diagnosis (McDonald 2010)			
CIS	18 (45%)	8 (20%)	3 (8.1%)
RRMS	22 (55%)	32 (80%)	30 (81%)
SPMS	0	0	3 (8.1%)
*Unknown*			*4*
Diagnosis considering OCB			
CIS	3		
RRMS	27		
SPMS	0		
*Unknown*			
Patients with contrast-enhancing lesions	8 (20%)	1 (2.5%)	0 (0%)
*Unknown*	*2*	*4*	*38*
Patients with relapse	19 (48%)	2 (5%)	0
Therapy			
High-efficacy treatment	6 (16%)	8 (22%)	11 (30%)
Low-efficacy treatment	32 (84%)	29 (78%)	26 (70%)
No	2	3	3

Values are depicted as mean (SD) or *n* (%). For EDSS, minimum and maximum values are also provided in squared brackets.

EDSS, Expanded Disability Status Scale; CIS, clinically isolated syndrome; OCB, oligoclonal band; RRMS, relapsing–remitting MS; SPMS, secondary progressive MS.

Lumbar puncture results for the assessment of OCB positivity for patients who were classified as CIS according to the 2010 McDonald criteria were collected to classify early MS patients also according to the recent criteria of 2024.^12^ Thus, 15 of 18 CIS patients were OCB positive, resulting in only three patients who remained classified as CIS.

### Choroid plexus volume

**Figure 2:**
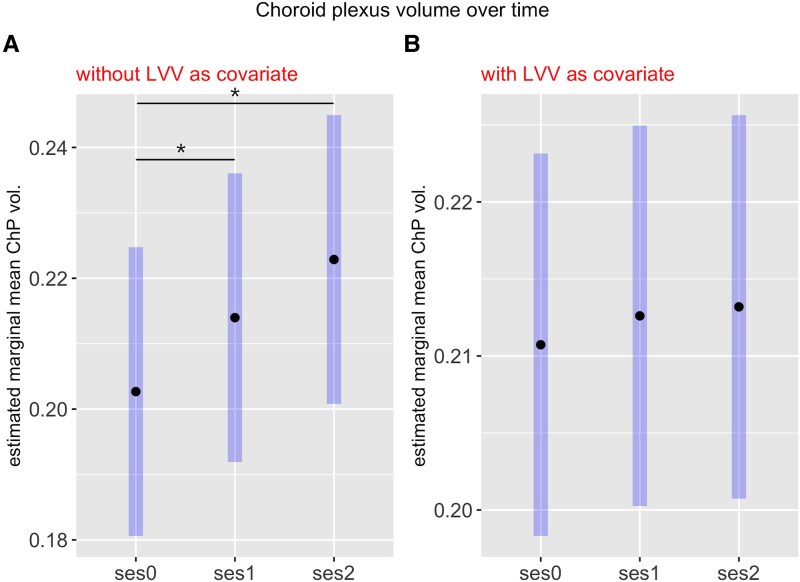
**Choroid plexus volume over time.** Results from the longitudinal analysis of choroid plexus volumes (normalized to intracranial volume, *N* = 40). Values were derived from linear mixed model analysis and represent estimated marginal means for baseline (ses0), follow-up after 2 years (ses1) and follow-up after 6–8 years (ses2) as bold dots and 95% confidence intervals as highlighted bars. Significant differences (*P* < 0.05) are marked with asterisks. (**A**) The analysis included age (at baseline) and sex as fixed effects and subject as random effect. (**B**) The analysis included age (at baseline), sex and LVV as fixed effects and subject as random effect.

Longitudinally, ChP volumes were significantly increased over the whole follow-up time from ses0 to ses2 [*P* = 0.0001, 95% confidence interval (CI): −0.03, −0.009] and within the first 2 years from ses0 to ses1 (*P* = 0.04; 95% CI, −0.02, −0.002) (Fig. 2A). However, longitudinal alterations were not significant after adding LVV as a covariate of no interest (*P* > 0.8 for each pairwise session comparison; Fig. 2B). Average annualized ChP volume change rates were 0.005 for the first follow-up period from ses0 to ses1 (ses1 relative to baseline: 5%) and 0.002 for the second period from ses1 to ses2 (ses2 relative to baseline: 7%), which was significantly different (paired *t*-test, *P* = 0.027; 95% CI, 3.9 × 10^−4^, 6.5 × 10^−3^; mean of differences, 3.4 × 10^−3^).

ChP volumes were not significantly associated with EDSS or EDSS progression. At each session, we found no significant relationship between ChP volumes and lesion load or lesion expansion calculated as the difference between two sessions. There was also no association between ChP volumes and the occurrence of relapses at baseline or contrast-enhancing lesions. ChP volumes and lesion load at each time point are summarized in Table 2.

**Table 2:** MRI measures for all three time points

	ses0 (baseline)	ses1 (2 years)	ses2 (6–8 years)
TLV [mL]	2.5 (1.1, 6.5)	2.0 (1.1, 4.9)	2.9 (1.1, 8.0)
Choroid plexus volume [ratio TIV × 100]	0.19 (0.14, 0.26)	0.20 (0.16, 0.27)	0.21 (0.16, 0.28)
T1w/FLAIR ratio			
NAWM	1.67 (1.63, 1.70)	1.68 (1.65, 1.71)	1.68 (1.64, 1.71)
Lesions	1.03 (0.95, 1.07)	0.99 (0.95, 1.06)	0.99 (0.93, 1.04)
Lesion rims	1.08 (1.02, 1.11)	1.05 (1.02, 1.10)	1.05 (1.00, 1.09)
Lesion centres	0.85 (0.78, 0.90)	0.81 (0.75, 0.89)	0.82 (0.74, 0.85)

Values are depicted as median (IQR) or *n* (%).

### T1-weighted/fluid-attenuated inversion recovery ratios

One baseline patient data had to be excluded due to lacking 3D-FLAIR data. T1w/FLAIR ratios were lower within lesions compared to NAWM and showed higher values in lesion rims compared to lesion centres (Table 2). An example for lesion rim and centre masks on T1w/FLAIR ratio map is visualized in the Supplementary material (Supplementary Fig. 4).

**Figure 3:**
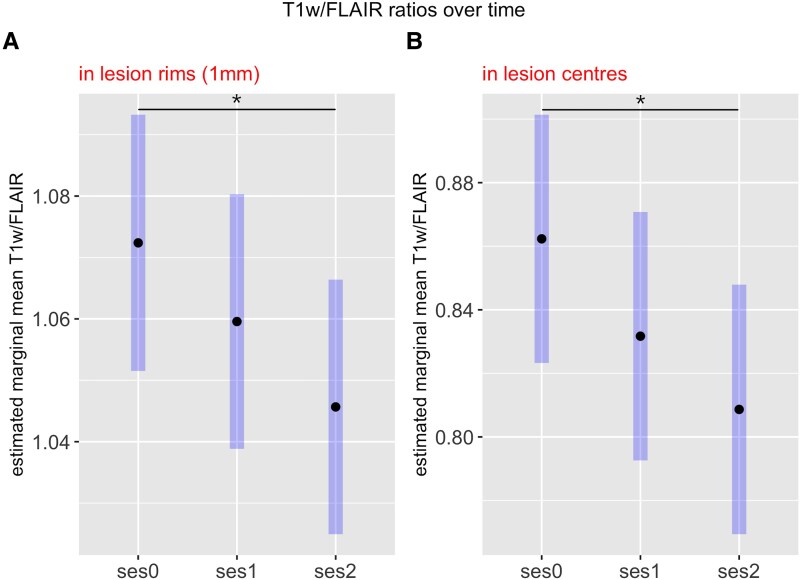
**T1w/FLAIR ratios over time.** Results from longitudinal analyses of T1w/FLAIR ratios in lesion rims (**A**) and lesion centres (**B**) (*N* = 39). Values were derived from linear mixed model analysis and represent estimated marginal means for baseline (ses0), follow-up after 2 years (ses1) and follow-up after 6–8 years (ses2) as bold dots and 95% confidence intervals as highlightedbars. The analysis included age (at baseline) and sex as fixed effects and subject as random effect. Significant differences (*P* < 0.05) are marked with asterisks.

T1w/FLAIR ratios were constant over time in NAWM, but were significantly decreased in lesion centres (*P* = 0.001; 95% CI, 0.02, 0.088) and rims (*P* = 0.02; 95% CI, 0.004, 0.049) over the whole follow-up time, i.e. between ses0 and ses2 (Fig. 3) From ses0 to ses1, the decrease did not reach significance. Erosion thresholds of 1.5 or 2 mm for the creation of lesion rims did not change the results (Supplementary Fig. 1).

At baseline, T1w/FLAIR ratios in both NAWM (*P* = 0.050) and rims of lesions (*P* = 0.048) were negatively associated with TLV, which remained significant at ses1 only for lesion rims (*P* = 0.01) but not for NAWM (*P* = 0.1). At ses2, none of them showed a significant relationship.

No significant relationship between T1w/FLAIR ratios and EDSS was found at each session. At baseline, T1w/FLAIR ratios within lesion rims and centres showed a significant negative association with disease duration (*P* = 0.005 for rims and *P* = 0.04 for centres).

### Choroid plexus volume and T1-weighted/fluid-attenuated inversion recovery ratios

**Figure 4:**
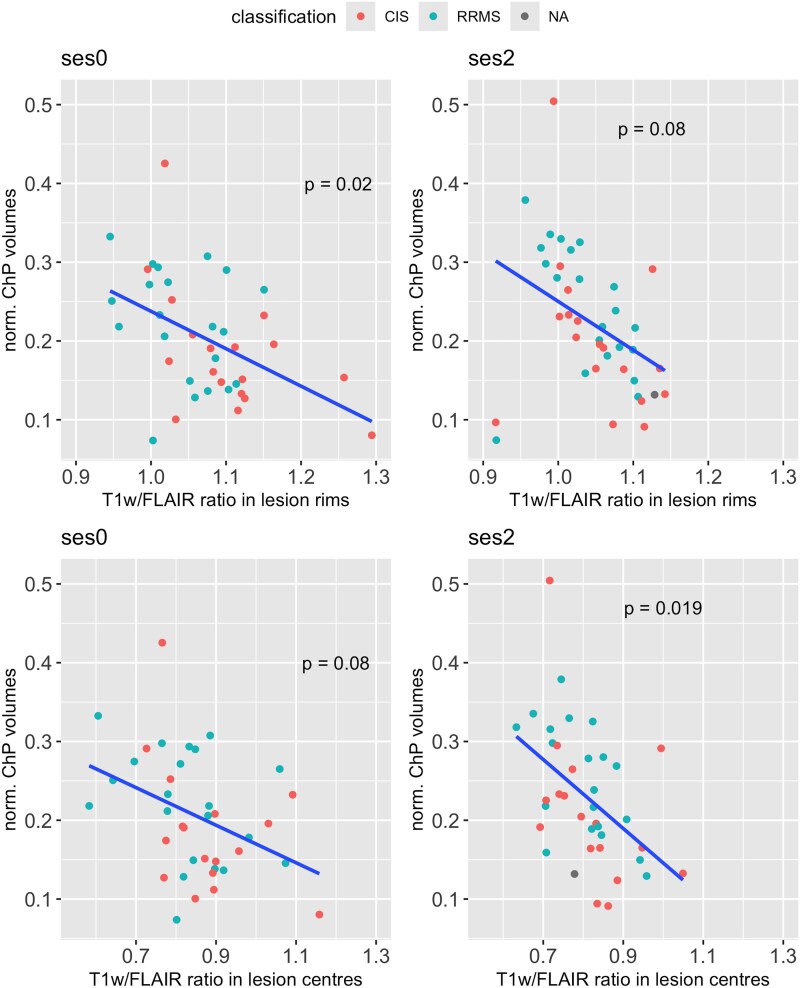
**Choroid plexus volume versus lesion T1w/FLAIR ratios.** Relationship between normalized ChP volumes and T1w/FLAIR ratios within lesion rims (upper row) or T1w/FLAIR ratios within lesion centres (lower row) at baseline (ses0) and at the last follow-up (ses2) (*N* = 39). Linear regression is represented by the bold line. *P*-values (for T1w/FLAIR ratio estimates) were derived from linear regression analyses including age (at baseline) and sex as covariates. CIS and RRMS patients (RRMS) are coloured in red and blue, respectively.

ChP volumes showed a significant relationship to T1w/FLAIR ratios in rims of lesions at sessions ses0 (*P* = 0.02; estimated beta = −0.36; model’s adj. R^2^ = 0.3) and to T1w/FLAIR ratios in lesion centres at session ses2 (*P* = 0.019; estimated beta = −0.31; model’s adj. R^2^ = 0.39) with age and sex as covariates (Fig. 4). No association was found within NAWM.

A summary of statistical parameters for linear mixed model and linear regression analyses is provided in the Supplementary material (Supplementary Tables 1 and 2).

### Comparison between clinically isolated syndrome and relapsing–remitting multiple sclerosis

**Figure 5:**
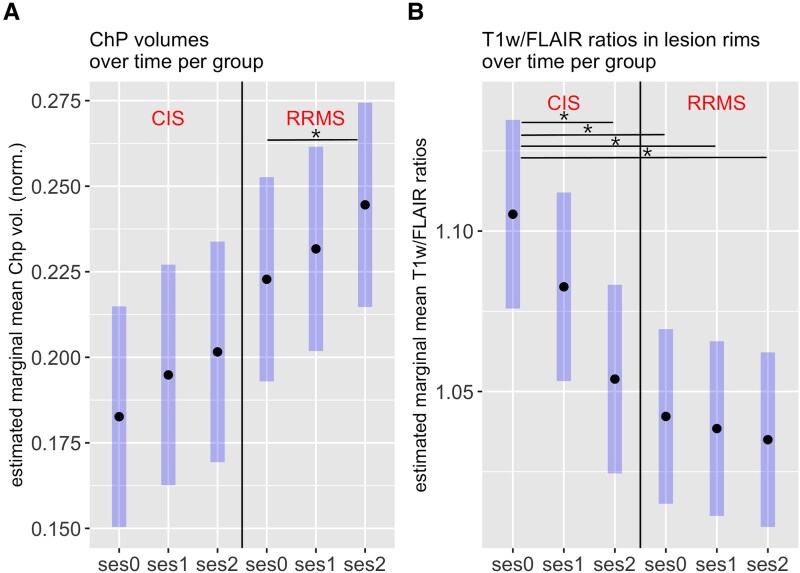
**Longitudinal biomarker patterns for CIS and RRMS patients.** Time courses of ChP volume (normalized to intracranial volume) (**A**) and T1w/FLAIR ratios in lesion rims (**B**) for CIS (*N* = 18, left panel) and RRMS (*N* = 22, right panel) patients. Values were derived from linear mixed model analysis and represent estimated marginal means for baseline (ses0), follow-up after 2 years (ses1) and follow-up after 6–8 years (ses2) as bold dots and 95% confidence intervals as highlighted bars. The analysis included age (at baseline) and sex as fixed effects and subject as random effect. Significant differences (*P* < 0.05) are marked with asterisks.

The subgroup analysis separating patients classified as CIS (*n* = 18) and RRMS (*n* = 22) at ses0 showed that the observed decrease in T1w/FLAIR ratios in lesions over time was not present in RRMS patients, but CIS patients showed a decrease over time for both rims (*P* = 0.004) and centres (*P* = 0.002) of lesions. Figure 5B shows the situation in lesion rims, which was similar for the lesion centres. Moreover, T1w/FLAIR ratios in lesion rims were higher for CIS at ses0 than RRMS at ses0, ses1 and ses2, with approximating the level of RRMS patients at ses2. T1w/FLAIR ratios in NAWM did not differ between both patient groups.

ChP volumes showed a rising gradient over time both in CIS and RRMS. We found a continuous increase in ChP in CIS from ses0 to ses2, which approached the ChP level of RRMS at ses0. In RRMS, ChP increased further between ses0 and ses2 (Fig. 5A). Although significance was not reached, a tendency towards higher ChP volume in CIS at ses2 compared to CIS at ses0 (*P* = 0.08; 95% CI, −0.0003, 1.4 × 10^−5^) and higher ChP volumes in RRMS at ses2 compared to CIS at ses0 (*P* = 0.07; 95% CI, −0.001, 3.18 × 10^−5^) was obtained. RRMS patients showed significantly higher ChP volumes at ses2 compared to ses0 (*P* = 0.01; 95% CI, −0.0004, −2.0 × 10^−5^).

The relationship between ChP volumes and T1w/FLAIR ratios was further examined for CIS and RRMS separately. This yielded a significant negative relationship for RRMS patients at ses2 in lesion centres (*P* = 0.007).

Lesion load was not different between CIS and RRMS. In CIS, no patient had a contrast-enhancing lesion at baseline, whereas all eight patients with gadolinium-positive lesions were RRMS. Both patient groups showed similar proportions of patients with or without a relapse within 3 weeks before the time of baseline MRI (52% had a relapse in RRMS; 39% had a relapse in CIS).

Since 83% of patients previously classified into CIS were OCB positive, we conducted a further subgroup analysis dividing patients into early and very early MS based on a disease duration at baseline of less (*n* = 24) or more than 6 months (*n* = 16). Very early MS patients showed lower ChP volumes than early MS patients, which were depicted in the Supplementary material (Supplementary Fig. 2A). As also for the CIS group, a tendency towards ChP increase between ses0 and ses2 in the very early MS group was found (*P* = 0.08). Moreover, a decrease in T1w/FLAIR ratios in lesion rims and centres was observed for the very early MS group rather than the early MS group (Supplementary Fig. 2B).

A summary of statistical parameters for each subgroup analysis is provided in the Supplementary material (Supplementary Tables 3 and 4).

## Discussion

In this longitudinal study of patients in the earliest stages of MS, we provided first evidence for a dynamic development of ChP volume increase in early MS over a time period of 6–8 years after diagnosis. This pointed to an ongoing long-term development rather than the attainment of a stable state. It might demonstrate that pathological processes that come along increased activity in the ChP are still ongoing during the course of the disease. This was independent of relapses or Gd-enhancing lesions at baseline MRI and not related to EDSS or disease progression. So far, only few studies analysed ChP changes over time, which analysed shorter follow-up periods compared to our study^23-27^ and most of them did not find significant changes over time. Klistorner *et al*.^23^ did not observe a significant increase in ChP volume over 4 years in patients with RRMS, but they later showed that ChP volume change was related to chronic lesion expansion and brain atrophy.^24^ Furthermore, an association between the increase in ChP volume after 5 years and blood-based neuronal injury markers was found.^25^ Another study found significantly increased ChP volumes and a concurrent impairment of glymphatic clearance measured by diffusion tensor imaging along the perivascular space (DTI-ALPS), showing a decreased DTI-ALPS index in RRMS patients over a period of 1–2 years.^26^ Bergsland *et al*.^27^ did not find an increase in ChP volumes or ChP inflammation measured by a T2 equivalent within the plexus over 5 years in a group of RRMS and progressive MS patients. All these studies suggested the importance of ChP enlargement for neurodegenerative processes and disease progression, but most of them did not find significant changes over time periods of less than 6 years. Possible explanations might include that short-term changes might be too small to detect or processes that lead to further increasing ChP volumes might show a more delayed effect. Technical differences for ChP segmentations must also be considered when comparing results of studies that have used different segmentation methods.

Since the volume of the ChP might be influenced by the ventricle volume due to mechanical enlargement, which was controversially discussed in previous studies,^28,29^ we conducted a second analysis adding also LVV as a covariate of no interest. The lack of significance for longitudinal changes over all time points highlighted the huge bias that might evolve from a physiological connection between ChP and lateral ventricles. Based on ChP volume alone, it might not be possible to draw conclusions about the underlying reasons for associations between ChP and ventricular enlargement, but previous studies suggest that ChP enlargement occurs early during disease when ventricle expansion was not yet detectable.^30,31^ In a recent study investigating T1w/T2w ratios within the ChP, pathological interrelations were also observed, but it was suggested that ventricle and ChP enlargement did not necessarily emerge in parallel.^32^ It might be also worth noting that, in other studies in which no longitudinal ChP enlargement was also found, the lateral ventricle was not considered in the statistical analysis.^27^

Paralleling those ChP changes, we demonstrated that tissue damage, as measured by T1w/FLAIR ratios, also followed a dynamic process of growing severity over time in T2w hyperintense MS lesions but not in NAWM. This lesional T1w/FLAIR decrease could be observed over the follow-up period in the entire MS group, indicating that even these early disease stages are characterized by increasing demyelination or loss of axonal integrity within lesions. Thus, T1w/FLAIR ratios within lesions might constitute an early marker for disease progression. In general, our T1w/FLAIR ratio results confirm previous findings that showed decreased T1w/T2w values in lesional tissue compared to NAWM.^33,34^ Lower T1w/FLAIR ratios are thought to correspond to more severe tissue damage representing demyelination, axonal loss or dendritic degradation.^9,33^ In our study, the observed tissue damage was more pronounced in lesion centres than in lesion rims since lowest T1w/FLAIR ratios were found within lesion centres in all patients. However, the general interpretation of T1w/FLAIR ratios is weakened by the lack of histopathological validation. So far, correlations with T1w/T2w ratios or MTR measures indicated that T1w/FLAIR ratios might provide similar aspects regarding tissue damage.^9^ Image ratios of T1w/T2w contrast were used to evaluate tissue damage and demyelination as these ratios were considered to reflect increased myelin contrast.^5,35^ In our own previous study, we found decreased T1w/FLAIR ratios in the NAWM of patients with MS showing a periventricular gradient, which provide evidence for representing tissue changes spreading from the CSF.^10^ Since this was observed also for MTR in other studies,^36-38^ similarities between both biomarkers are reasonable. Another study investigated the microstructure of chronic MS lesions by T1w/FLAIR ratios and observed more severe damage in iron rim lesions compared to non-iron rim lesions.^39^ Although T1w/FLAIR ratios appear to be a promising biomarker, future studies should consider potentials for standardization, since scanner and sequence variabilities could have an impact on ratio outcomes. In the present study, scans were derived from a standardized protocol from a single scanner, which hampered generalizability, on the one hand, and reduced potential biases, on the other hand.

We demonstrated that the enlargement of the ChP correlated with reduced T1w/FLAIR ratios in MS lesions. Specifically, this correlation was evident at diagnosis (ses0) at the lesions’ rims and after 6–8 years (ses2) at the lesions’ centre. This provided evidence that pathological mechanisms occurring in the ChP could be relevant for lesional tissue damage. However, our data do not allow us to draw final conclusions about the nature of the pathological processes causing the association between plexus volume and lesion pathology, and we cannot determine whether the effects are causally related or merely run parallel. Still, previously published studies suggested that the ChP could be involved in processes at the edges of chronic lesions since ChP was shown to be larger in patients with iron rim lesions.^40^ Additionally, according to recent published studies, a link between both structures seems plausible on the basis of immune activation, which has been shown for the ChP-CSF barrier,^41^ activated microglia and macrophages at the lesion edge and demyelinated lesion centres of chronic active lesions.^42^ Previously, a high prevalence of smouldering chronically active lesions based on their innate immune cell content has been shown in periventricular areas in MS patients with relapsing or progressive disease types.^43^ In that study, lesions have been classified into homogeneously active, rim-active or non-active lesions characterized by either active lesion centre, active lesion rims or inactive centre and rims, respectively, measured by [18F]-DPA-714 uptake.^43^ Therein, an association between ChP volumes and the percentage of homogeneously active lesions in the vicinity of the ventricular borders was found, indicating that ChP dysfunction could contribute to the persistence of pro-inflammatory microglial activation.^43^ Further, the failure of myelin repair in periventricular lesions has been shown to be related to ChP enlargement.^38^ In our study, contrasting the cited works, our patients were at a very early state of their disease the time of inclusion with relatively low lesion loads, but typically with a high prevalence of lesions in periventricular areas. Thus, our results of a longitudinal decrease in T1w/FLAIR ratios within lesion centres and rims could also point to reduced ability of remyelination. The concurrent association with ChP enlargement fitted to the hypothesis that ChP-based microglial activation and the failure of myelin repair in lesions are connected and contribute to neurodegeneration.

Differentiation between patients who were either classified as RRMS or CIS at study entry provided a more detailed insight into early ChP enlargement and lesion pathology, although the resulting subgroups were small. In addition, nearly all of our patients who were classified as CIS at baseline according to the McDonald criteria of 2010 converted to RRMS during follow-up. According to the current diagnosis criteria considering OCB positivity, they could be also referred to a very early RRMS group, who had a disease duration of ≤6 months at study inclusion. We demonstrated constant increases of ChP volumes over time in both subgroups, with overall higher levels in the RRMS group, pointing to ongoing ChP activation in both subgroups over the follow-up period. In contrast, regarding T1w/FLAIR, a decrease over time was evident within lesion rims and centres only in the CIS group, approaching the lower T1w/FLAIR levels of RRMS patients, which in turn showed a stable state over time. We draw the conclusion that a higher but constant degree of lesion pathology was present in the RRMS group already at baseline, which was gradually attained in the CIS group only after 6–8 years of disease duration. A relationship between ChP volumes and T1w/FLAIR ratios within lesion centres at ses2 was only significant in RRMS patients, which highlighted, not surprisingly, that the strongest effects of lesion destructivity in lesion centres are present in later stages.

Due to outdated classification criteria, a more in-depth analysis of the patient groups was conducted by considering also their OCB positivity as it would also be done based on the current diagnostic criteria.^12^ Since most CIS patients were then already be classified as RRMS, we additionally divided our patient group based on their disease duration, which we assumed to provide similar results as our CIS group was characterized by shorter disease durations than our RRMS group. The very early MS group then showed a decrease in T1w/FLAIR ratios within lesions over time as previously observed for the CIS group. However, no group differences between very early and early MS between the three time points were found. Regarding ChP enlargement, the very early MS group was characterized by lower ChP volume at each session compared to the early MS group, indicating similar results compared to the CIS/RRMS classification, which were, however, not significant before. A tendency towards longitudinal ChP enlargement was found for the very early MS group as previously found for the CIS group. Overall, our results indicated similarities in longitudinal patterns between both subgroup methods, indicating that previous outdated diagnostic classifications could be approximated by the use of disease duration, which might be the reason for the observed differences between CIS and RRMS patients.

Regarding the relationship to disease progression as measured by the EDSS, we were not able to show associations between ChP volumes at baseline and EDSS at follow-up or associations with EDSS progression during this follow-up period. In general, previous studies found inconsistent results for the association between ChP and EDSS on a cross-sectional level, but none of these investigated ChP enlargement and future EDSS worsening, as measured by the difference between baseline and follow-up EDSS, so far. Thus, one study found no significant correlation between ChP volumes and EDSS in RRMS,^44^ whereas Storelli *et al*.^45^ showed an association. Fleischer *et al*.^4^ found a relationship between ChP volume and EDSS scores at baseline and at 4 years of follow-up in a group of CIS and MS patients. In our study, patients showed a relatively stable and low EDSS, which might make it difficult to identify a relationship to EDSS progression. Overall, our cohort was characterized not only by very early disease stages at study entry but also by short monitoring intervals, which made patients benefit from best possible treatment possibilities and optimal medication doses. Therefore, it is likely that our cohort represents a group of patients with minimal EDSS changes. As such, the contribution of dynamic ChP volume changes to disability progression over time remains unclear.

In contrast to our previous study, we were not able to show an association between ChP volume and lesion load.^10^ This might be due to a smaller sample size and lower lesion loads in the present study sample. Other studies found relationships between enlarged ChP and greater lesion volumes,^40,46^ but its detailed pathological mechanism remains unknown. However, lesion load is not solely related to active inflammation as it represents a cumulative indication of tissue damage. Therefore, we could not conclude based on these results if there is a stronger relationship with inflammation than with tissue damage or vice versa. In-depth regional analyses of distinct lesion pathologies would be necessary to further elucidate this association.

Some additional limitations have to be considered in this study. First, we did not compare our patients’ results to healthy controls; instead, we concentrated on the longitudinal evolution of ChP and T1w/FLAIR and regarded the baseline values as internal references. However, one of the study’s main aspect regarding T1w/FLAIR ratios in lesions does not require the inclusion of control data as the amount of lesion is negligible. Second, the tissue class of lesion rims might also include very small lesions, which did not survive the eroding step, or it might include voxels of NAWM or pre-lesional tissue due to partial volume effects. In that context, lesion rims might be affected by differences between T1w and FLAIR contrasts at which lesion sizes are depicted differently so that lesions on FLAIR might be represented larger than on T1w. This effect could lead to an overestimation of T1w/FLAIR in those areas and would thus weaken the effects and associations we have investigated. Third, the influence of the lateral ventricle volume on the ChP volume is still not clarified, although we showed that results changed when considering it as a covariate. However, this only showed pathological interrelation and not their common mechanisms as also suggested in a previous study.^32^ It might be worth to investigate if microstructural alterations within the ChP might be more independent than ChP volume from ventricular enlargement. Fourth, we were not able to conduct a subgroup analysis with those CIS patients who convert to RRMS due to limited sample size. Similarly, the subgroup analysis comparing CIS and RRMS patients should be interpreted with caution due to the small sample sizes and the usage of outdated classification criteria. In addition, missing values in the longitudinal analyses could introduce a bias, but missing values were only present for EDSS at ses2, so that this concerns only a minor part of our results, and subjects with missing values laid on group averages at baseline (EDSS 2.0 and 1.0). Fifth, longitudinal analyses of any volumetric measure could generally be influenced by biases due to ageing or scanner drifts that emerge during time.

In summary, we found longitudinal changes of both ChP enlargement and T1w/FLAIR ratios over 6–8 years in early MS patients. The ChP seemed to be associated with T1w/FLAIR ratios within lesion rims at baseline and within lesion centres at the last follow-up, indicating that ChP enlargement is involved in the dynamic development of lesion destructivity starting from the edges to the centre.

## Supplementary Material

fcag129_Supplementary_Data

## Data Availability

Raw MRI and clinical data cannot be made available due to data protection regulations. The pipeline used for the creation of T1w/FLAIR ratio maps is publicly available at https://github.com/treanus/KUL_NIS. For choroid plexus segmentation, we used a publicly available toolbox (https://github.com/Center-of-Imaging-Biomarker-Development/chp_seg).
