# Supplementary material for: Longitudinal changes of choroid plexus volumes and MRI ratios in multiple sclerosis
Source: Brain Commun. 2026 Apr 18;8(3):fcag129. doi: 10.1093/braincomms/fcag129 (PMC13180650; doi:10.1093/braincomms/fcag129)
Supplement: fcag129_Supplementary_Data [file fcag129_supplementary_data.pdf]

Supplementary Materials

1. Summary of statistical parameters

Supplementary Table 1 Results for linear mixed model analyses.

Purpose	covariates	pairwise comparisons	estimate	p-value [95% CI]
Longitudinal ChP vol.	age (at baseline) and sex as fixed effects; subject as random effect	ses0 – ses1<br>ses0 – ses2<br>ses1 – ses2	-0.011<br>-0.02<br>-0.009	0.044 [-0.02,-0.002]<br>0.0001 [-0.03,-0.009]<br>0.14 [-0.02,0.002]
Longitudinal ChP vol.	age (at baseline), sex and LVV as fixed effects; subject as random effect	ses0 – ses1<br>ses0 – ses2<br>ses1 – ses2	-0.0019<br>-0.002<br>-0.0006	0.86 [-0.01,0.007]<br>0.80 [-0.01,0.007]<br>0.99 [-0.009,0.008]
Longitudinal T1w/FLAIR ratios (lesion rims)	age (at baseline) and sex as fixed effects; subject as random effect	ses0 – ses1<br>ses0 – ses2<br>ses1 – ses2	0.013<br>0.027<br>0.014	0.37 [-0.0098,0.036]<br>0.02 [0.004,0.049]<br>0.31 [-0.009,0.036]
Longitudinal T1w/FLAIR ratios (lesion centres)	age (at baseline) and sex as fixed effects; subject as random effect	ses0 – ses1<br>ses0 – ses2<br>ses1 – ses2	0.03<br>0.05<br>0.02	0.08 [-0.003,0.064]<br>0.001 [0.02,0.088]<br>0.24 [-0.011,0.057]

Supplementary Table 2 Results for linear regression analyses.

Purpose		adj. R^2 (p-value)	Coefficients	estimated beta	p-values
ChP vol. vs. EDSS	ses0	0.76 (< 0.0001)	age	0.002	0.73
			sex	0.008	0.55
			LVV	0.08	< 0.0001
			EDSS	0.003	0.60
	ses1	0.76 (< 0.0001)	age	0.007	0.18
			sex	0.01	0.44
			LVV	0.06	< 0.0001
			EDSS	0.001	0.82
	ses2	0.80 (< 0.0001)	age	0.007	0.27
			sex	0.004	0.83
			LVV	0.06	< 0.0001
			EDSS	0.004	0.48
ChP vol. vs. EDSS progression	ses0 to ses2	0.75 (< 0.0001)	age	0.0002	0.71
			sex	0.008	0.62
			LVV	0.08	< 0.0001
			EDSS progr.	-0.00004	0.99
ChP vol. vs. TLV	ses0	0.76 (< 0.0001)	age	0.0002	0.7
			sex	0.007	0.58
			LVV	0.075	< 0.0001
			TLV	0.00007	0.96
	ses1	0.76 (< 0.0001)	age	0.0009	0.15
			sex	0.01	0.44
			LVV	0.06	< 0.0001
			TLV	-0.0007	0.7
	ses2	0.81 (< 0.0001)	age	0.0009	0.13
			sex	0.006	0.69
			LVV	0.06	< 0.0001
			TLV	-0.001	0.48
ChP vol. vs. dd	ses0	0.76 (< 0.0001)	age	0.0002	0.76
			sex	0.011	0.43

			LVV	0.08	< 0.0001
			dd	-0.009	0.42
T1w/FLAIR ratios (lesion rims) vs. TLV	ses0	0.15 (0.03)	age	-0.002	0.08
			sex	-0.01	0.61
			TLV	-0.005	0.048
	ses1	0.17 (0.02)	age	-0.0007	0.46
			sex	-0.008	0.70
			TLV	-0.008	0.01
	ses2	0.03 (0.24)	age	-0.0008	0.33
			sex	0.000009	0.99
			TLV	-0.003	0.24
T1w/FLAIR ratios (lesion centres) vs. TLV	ses0	0.09 (0.09)	age	-0.004	0.04
			sex	-0.03	0.57
			TLV	-0.005	0.33
	ses1	0.07 (0.14)	age	-0.002	0.26
			sex	-0.026	0.57
			TLV	-0.009	0.12
	ses2	0.05 (0.2)	age	-0.002	0.23
			sex	-0.0001	0.99
			TLV	-0.004	0.25
T1w/FLAIR ratios (lesion rims) vs. EDSS	ses0	0.05 (0.18)	age	-0.002	0.03
			sex	-0.009	0.71
			EDSS	0.0004	0.97
	ses1	0.002 (0.39)	age	-0.001	0.15
			sex	0.003	0.91
			EDSS	-0.003	0.78
	ses2	-0.0095 (0.46)	age	-0.0008	0.38
			sex	0.008	0.71
			EDSS	-0.005	0.51
T1w/FLAIR ratios (lesion centres) vs. EDSS	ses0	0.07 (0.14)	age	-0.004	0.03
			sex	-0.03	0.58
			EDSS	-0.008	0.71
	ses1	-0.0005 (0.41)	age	-0.003	0.10
			sex	-0.014	0.77
			EDSS	0.003	0.87
	ses2	0.02 (0.31)	age	-0.001	0.40
			sex	0.012	0.72
			EDSS	-0.01	0.27
T1w/FLAIR ratios (lesion rims) vs. dd	ses0	0.087 (0.11)	age	-0.002	0.034
			sex	-0.003	0.9
			dd	-0.02	0.27
T1w/FLAIR ratios (lesion centres) vs. dd	ses0	0.10 (0.08)	age	-0.004	0.02
			sex	-0.012	0.80
			dd	-0.04	0.25
ChP vol. vs. T1w/FLAIR ratios (lesion rims)	ses0	0.3 (0.001)	age	0.002	0.09
			sex	-0.03	0.15
			T1w/FLAIR	-0.36	0.02
	ses1	0.30 (0.001)	age	0.002	0.01
			sex	-0.03	0.15
			T1w/FLAIR	-0.22	0.16
	ses2	0.39 (0.0001)	age	0.003	0.005
			sex	-0.05	0.06
			T1w/FLAIR	-0.39	0.07
ChP vol. vs. T1w/FLAIR ratios (lesion centres)	ses0	0.25 (0.004)	age	0.002	0.07
			sex	-0.03	0.17
			T1w/FLAIR	-0.14	0.08
	ses1	0.28 (0.002)	age	0.002	0.02
			sex	-0.03	0.14
			T1w/FLAIR	-0.13	0.10

	ses2	0.39 (0.0002)	age	0.002	0.02
			sex	-0.04	0.09
			T1w/FLAIR	-0.31	0.02

‘Age’ indicates age at baseline; ChP = choroid plexus, EDSS = Expanded disability status scale; dd = disease duration; LVV = lateral ventricle volume; TLV = total lesion volume

2. Freesurfer’s quality assurance tool

The quality assurance tool supplied in FreeSurfer for assessment of consistency of WM/GM contrast signal-to-noise (conSNR) ratio in the brain between the three time points. Therein, average GM/WM conSNR across all patients was stable at each time point (coefficients of variations: 3.3 ± 6.3%, 3.3 ± 5.6%, 3.3 ± 6.2% at ses0, ses1 and ses2 respectively). The variability between the time points (average of absolute differences divided by the mean conSNR of the time points) was well below 8% for each patient, showing a good consistency of image quality between the time points.

3. Erosion thresholds for rim mask creation

For the main analysis, lesion masks were eroded by 1 mm to receive lesion rim masks. Robustness of results were assessed by applying different erosion thresholds of 1.5 mm and 2 mm. With larger erosion thresholds, T1w/FLAIR ratios were decreasing and the results of longitudinal decrease in T1w/FLAIR ratios remained significant using each threshold (see Figure 1 below). Moreover, the association between ChP volume and T1w/FLAIR ratios was significant for ses0 for each erosion threshold (for 1.5mm: $p = 0.038$, estimated beta = -0.29, model’s adj. $R^2 = 0.28$, for 2mm: $p = 0.048$, estimated beta = -0.26, model’s adj. $R^2 = 0.27$).

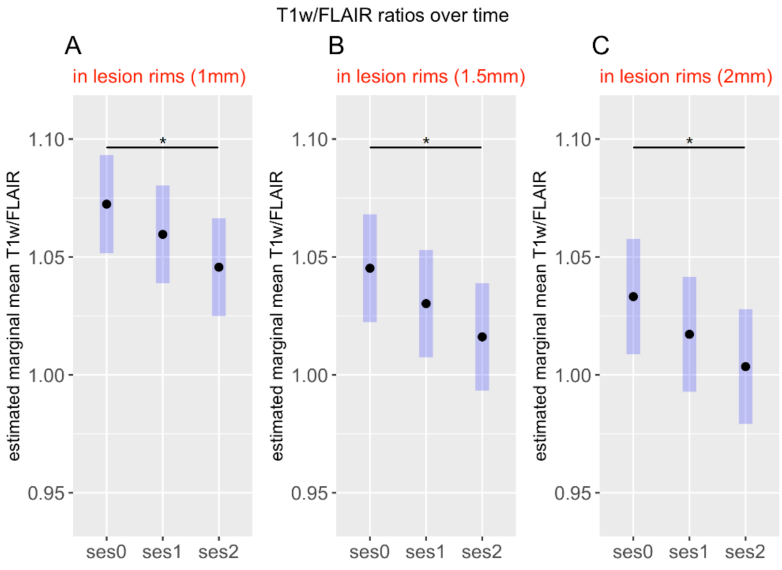

Supplementary Figure 1 Longitudinal changes of T1w/FLAIR ratios in lesion rims for different erosion thresholds. Values were derived from linear mixed model analysis and represent estimated marginal means for baseline (ses0), follow-up after two years (ses1), and follow-up after 6 to 8 years (ses2) (N = 39), and 95% confidence intervals as blue bars. The analysis included age (at baseline) and sex as fixed effects and subject as random effect. Significant differences ($p < 0.05$) are marked with asterisks. A: T1w/FLAIR ratios in 1 mm lesion rims over time. B: T1w/FLAIR ratios in 1.5 mm lesion rims over time. C: T1w/FLAIR ratios in 2 mm lesion rims over time.

4. Results from subgroup analysis CIS vs. RRMS

Supplementary Table 3 Results from subgroup analysis based on diagnosis from McDonald criteria 2010.

pairwise comparison	estimate (standard error)	p-value	95% confidence interval
**ChP volume**			
ses0 CIS – ses1 CIS	-0.012 (0.007)	0.501	-0.033, 0.008
ses0 CIS – ses2 CIS	-0.019 (0.007)	0.083	-0.039, 0.001
ses0 CIS – ses0 RRMS	-0.04 (0.022)	0.449	-0.105, 0.025
ses0 CIS – ses1 RRMS	-0.049 (0.022)	0.237	-0.114, 0.016
ses0 CIS – ses2 RRMS	-0.062 (0.022)	0.070	-0.127, 0.003
ses1 CIS – ses2 CIS	-0.007 (0.007)	0.927	-0.027, 0.014
ses1 CIS – ses0 RRMS	-0.028 (0.022)	0.791	-0.093, 0.037
ses1 CIS – ses1 RRMS	-0.037 (0.022)	0.544	-0.102, 0.028
ses1 CIS – ses2 RRMS	-0.05 (0.022)	0.224	-0.115, 0.015
ses2 CIS – ses0 RRMS	-0.021 (0.022)	0.923	-0.086, 0.044
ses2 CIS – ses1 RRMS	-0.03 (0.022)	0.736	-0.095, 0.035
ses2 CIS – ses2 RRMS	-0.043 (0.022)	0.374	-0.108, 0.022
ses0 RRMS – ses1 RRMS	-0.009 (0.006)	0.739	-0.028, 0.01
ses0 RRMS – ses2 RRMS	-0.022 (0.006)	0.014	-0.041, -0.003
ses1 RRMS – ses2 RRMS	-0.013 (0.006)	0.353	-0.032, 0.006
**T1w/FLAIR ratios in lesion rims**			
ses0 CIS – ses1 CIS	0.023 (0.014)	0.569	-0.018, 0.063
ses0 CIS – ses2 CIS	0.051 (0.014)	0.005	0.011, 0.092
ses0 CIS – ses0 RRMS	0.063 (0.02)	0.029	0.004, 0.122
ses0 CIS – ses1 RRMS	0.067 (0.02)	0.017	0.008, 0.126
ses0 CIS – ses2 RRMS	0.07 (0.02)	0.011	0.011, 0.129
ses1 CIS – ses2 CIS	0.029 (0.014)	0.300	-0.011, 0.069
ses1 CIS – ses0 RRMS	0.04 (0.02)	0.346	-0.019, 0.099
ses1 CIS – ses1 RRMS	0.044 (0.02)	0.251	-0.015, 0.103
ses1 CIS – ses2 RRMS	0.048 (0.02)	0.181	-0.011, 0.107
ses2 CIS – ses0 RRMS	0.012 (0.02)	0.992	-0.047, 0.071
ses2 CIS – ses1 RRMS	0.015 (0.02)	0.972	-0.044, 0.074
ses2 CIS – ses2 RRMS	0.019 (0.02)	0.934	-0.04, 0.078
ses0 RRMS – ses1 RRMS	0.004 (0.013)	1.000	-0.033, 0.041
ses0 RRMS – ses2 RRMS	0.007 (0.013)	0.993	-0.03, 0.044
ses1 RRMS – ses2 RRMS	0.003 (0.013)	1.000	-0.034, 0.041

5. Further subgroup analysis based on disease duration at baseline

Supplementary Table 4 Results from subgroup analysis based on disease duration at baseline (very early MS: less than 6 months, early MS: more than 6 months).

pairwise comparison	estimate (standard error)	p-value	95% confidence interval
**ChP volume**			
ses0 very early MS – ses1 very early MS	-0.013 (0.006)	0.297	-0.03, 0.005
ses0 very early MS – ses2 very early MS	-0.016 (0.006)	0.080	-0.034, 0.001
ses0 very early MS – ses0 early MS	-0.053 (0.021)	0.130	-0.115, 0.009
ses0 very early MS – ses1 early MS	-0.063 (0.021)	0.047	-0.125, -0.001
ses0 very early MS – ses2 early MS	-0.079 (0.021)	0.006	-0.141, -0.017
ses1 very early MS – ses2 very early MS	-0.004 (0.006)	0.988	-0.021, 0.014
ses1 very early MS – ses0 early MS	-0.041 (0.021)	0.383	-0.103, 0.021
ses1 very early MS – ses1 early MS	-0.05 (0.021)	0.176	-0.112, 0.012
ses1 very early MS – ses2 early MS	-0.067 (0.021)	0.029	-0.129, -0.005
ses2 very early MS – ses0 early MS	-0.037 (0.021)	0.491	-0.099, 0.025
ses2 very early MS – ses1 early MS	-0.046 (0.021)	0.247	-0.108, 0.016
ses2 very early MS – ses2 early MS	-0.063 (0.021)	0.046	-0.125, -0.001
ses0 early MS – ses1 early MS	-0.009 (0.007)	0.795	-0.031, 0.012
ses0 early MS – ses2 early MS	-0.026 (0.007)	0.009	-0.047, -0.005
ses1 early MS – ses2 early MS	-0.017 (0.007)	0.225	-0.038, 0.005
**T1w/FLAIR ratios in lesion rims**			
ses0 very early MS – ses1 very early MS	0.025 (0.012)	0.299	-0.01, 0.061
ses0 very early MS – ses2 very early MS	0.042 (0.012)	0.010	0.007, 0.078
ses0 very early MS – ses0 early MS	0.041 (0.021)	0.406	-0.022, 0.103
ses0 very early MS – ses1 early MS	0.036 (0.021)	0.557	-0.027, 0.098
ses0 very early MS – ses2 early MS	0.045 (0.021)	0.300	-0.018, 0.108
ses1 very early MS – ses2 very early MS	0.017 (0.012)	0.710	-0.018, 0.052
ses1 very early MS – ses0 early MS	0.015 (0.021)	0.979	-0.047, 0.078
ses1 very early MS – ses1 early MS	0.01 (0.021)	0.997	-0.052, 0.073
ses1 very early MS – ses2 early MS	0.019 (0.021)	0.941	-0.043, 0.082
ses2 very early MS – ses0 early MS	-0.002 (0.021)	1.000	-0.064, 0.061
ses2 very early MS – ses1 early MS	-0.007 (0.021)	1.000	-0.069, 0.056
ses2 very early MS – ses2 early MS	0.002 (0.021)	1.000	-0.06, 0.065
ses0 early MS – ses1 early MS	-0.005 (0.015)	0.999	-0.048, 0.038
ses0 early MS – ses2 early MS	0.004 (0.015)	1.000	-0.039, 0.047
ses1 early MS – ses2 early MS	0.009 (0.015)	0.988	-0.033, 0.052

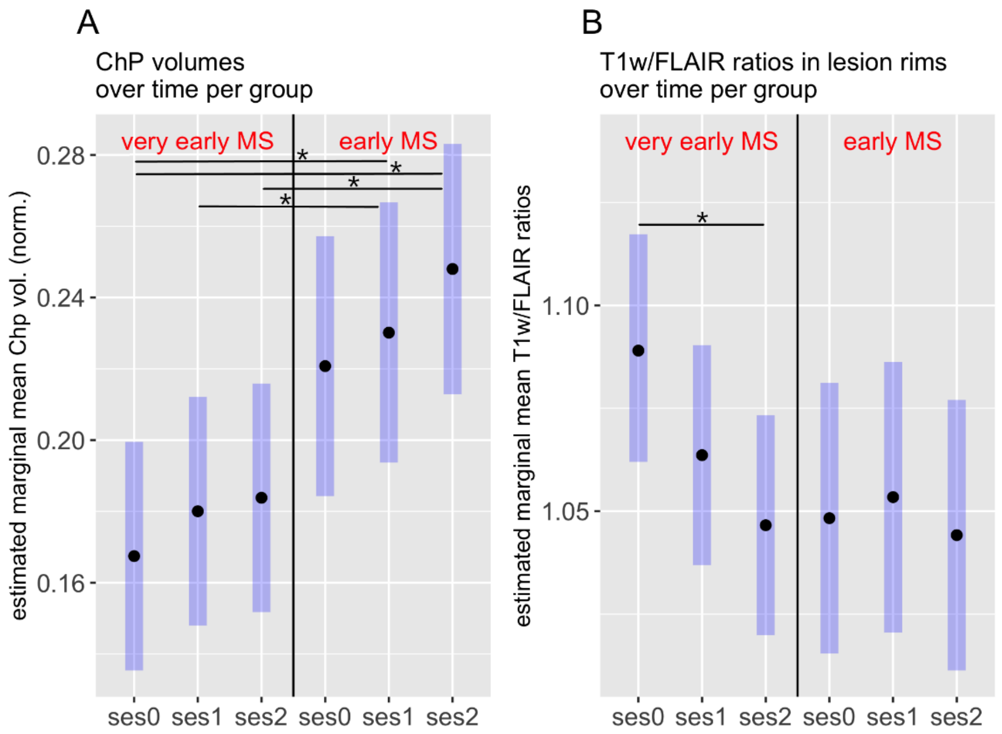

Supplementary Figure 2 Longitudinal changes of choroid plexus volumes and T1w/FLAIR ratios for very early and early MS groups. Values were derived from linear mixed model analysis and represent estimated marginal means for baseline (ses0), follow-up after two years (ses1), and follow-up after 6 to 8 years (ses2) and 95% confidence intervals as blue bars. The analysis included age (at baseline) and sex as fixed effects and subject as random effect. Significant differences ($p < 0.05$) are marked with asterisks. A: Choroid plexus (ChP) volumes over time in very early (left panel, N = 24) and early MS (right panel, N = 16). B: T1w/FLAIR ratios in lesion rims over time in very early (left panel, N = 23) and early MS (right panel, N = 16).

6. Supplementary Figures

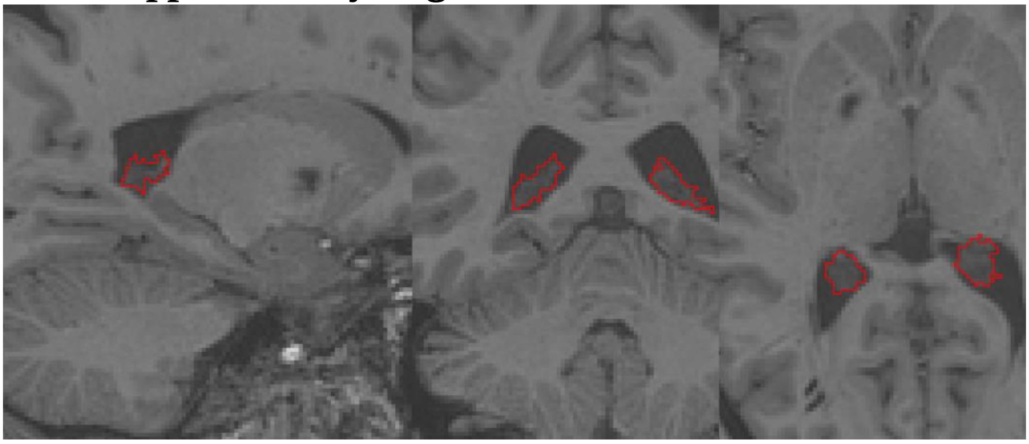

Supplementary Figure 3 Example for choroid plexus segmentation. Choroid plexus segmentation is visualised in red and overlaid on T1-weighted image.

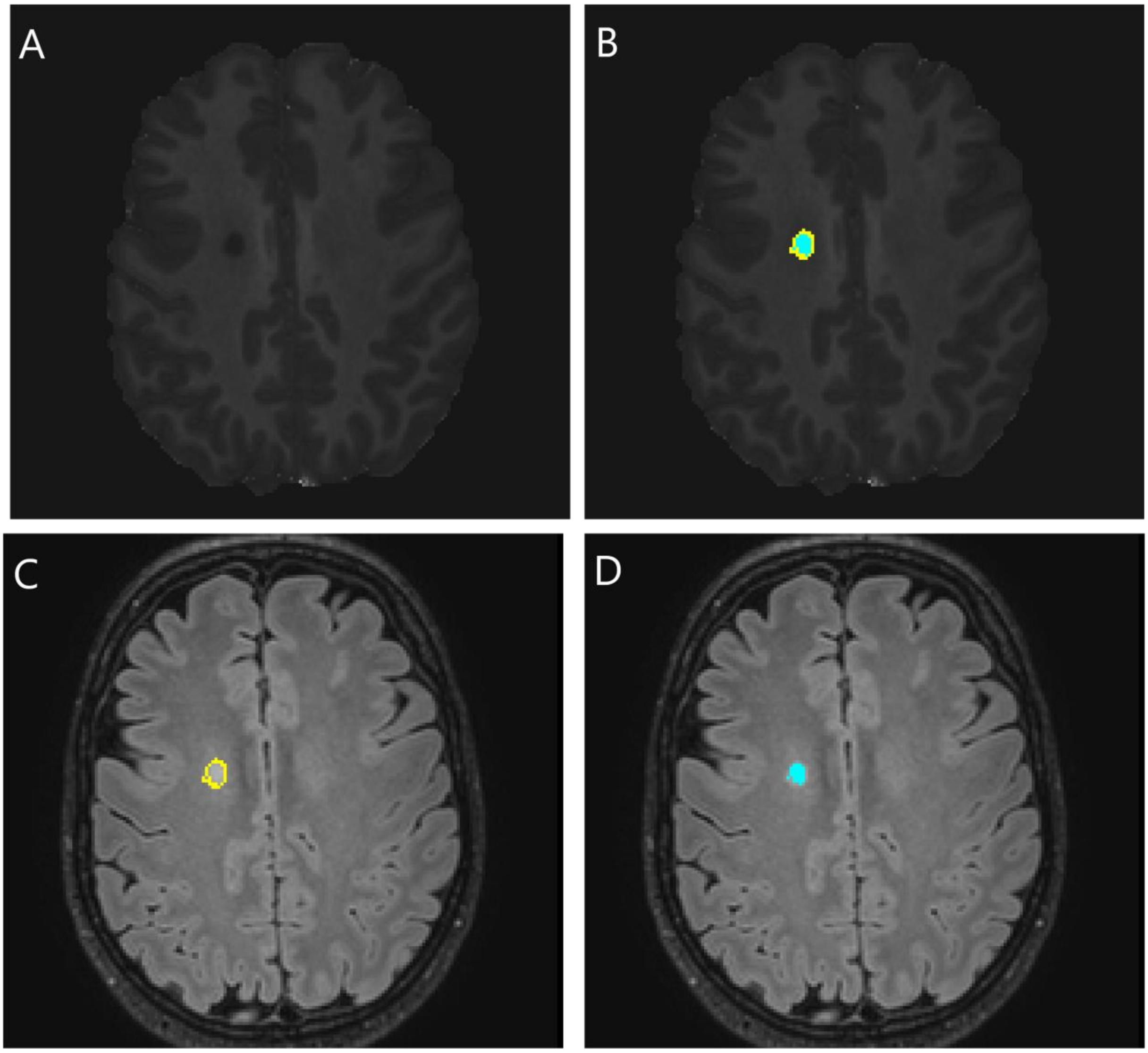

Supplementary Figure 4 Example of lesion rim and centre masks. A: T1w/FLAIR ratio map. B: Lesion rim (yellow) and centre (blue) masks overlaid on T1w/FLAIR ratio map. C: Lesion rim mask (yellow) overlaid on FLAIR image. D: Lesion centre mask (blue) overlaid on FLAIR image.
